# The Architecture of the Rag GTPase Signaling Network

**DOI:** 10.3390/biom7030048

**Published:** 2017-06-30

**Authors:** Raffaele Nicastro, Alessandro Sardu, Nicolas Panchaud, Claudio De Virgilio

**Affiliations:** 1 Department of Biology, University of Fribourg, Chemin du Musée 10, CH-1700 Fribourg, Switzerland; raffaele.nicastro2@unifr.ch (R.N.); alessandro.sardu@unifr.ch (A.S.); 2 Novartis Institutes for Biomedical Research, NIBR, Novartis Pharma AG, 4002 Basel, Switzerland; nicolas_panchaud@hotmail.com

**Keywords:** Rag GTPases, EGO complex, target of rapamycin complex 1 (TORC1), amino acid signaling, budding yeast, SEACIT, SEACAT, Lst4–Lst7

## Abstract

The evolutionarily conserved target of rapamycin complex 1 (TORC1) couples an array of intra- and extracellular stimuli to cell growth, proliferation and metabolism, and its deregulation is associated with various human pathologies such as immunodeficiency, epilepsy, and cancer. Among the diverse stimuli impinging on TORC1, amino acids represent essential input signals, but how they control TORC1 has long remained a mystery. The recent discovery of the Rag GTPases, which assemble as heterodimeric complexes on vacuolar/lysosomal membranes, as central elements of an amino acid signaling network upstream of TORC1 in yeast, flies, and mammalian cells represented a breakthrough in this field. Here, we review the architecture of the Rag GTPase signaling network with a special focus on structural aspects of the Rag GTPases and their regulators in yeast and highlight both the evolutionary conservation and divergence of the mechanisms that control Rag GTPases.

## 1. Introduction

Eukaryotic cell growth and metabolism are controlled and coordinated by various signaling pathways that can sense, propagate, and induce responses to environmental signals such as growth factors, hormones, and nutrients. In this context, a central and particularly well-studied pathway is the target of rapamycin (TOR) signaling pathway. The TOR proteins are serine/threonine protein kinases and members of the phosphatidylinositol 3-kinase-related kinase family of proteins that have been discovered in the budding yeast *Saccharomyces cerevisiae* due to the isolation of mutations, which rendered cells resistant to the macrolide rapamycin [1,2,3,4]. Parallel studies in mammalian cells revealed that the TOR proteins are highly conserved among eukaryotes [5,6,7,8]. They function in two structurally distinct protein complexes coined target of rapamycin complex 1 (TORC1) and TORC2 that regulate different aspects of cellular physiology [9,10]. While TORC1 regulates growth and metabolism through diverse anabolic and catabolic processes including the biosynthesis of proteins, lipids, and nucleotides, the biogenesis of ribosomes, and autophagy, TORC2 controls cell proliferation, survival, membrane tension, and turgor as further reviewed elsewhere [11,12,13,14]. Interestingly, TORC1, but not TORC2, is acutely sensitive to rapamycin when in complex with the proline isomerase Fpr1 in yeast (or FKBP12 in mammalian cells) [9], which can be elegantly explained by the fact that the rapamycin-Fpr1/FKBP12-binding domain in TORC2 is masked by a TORC2-specific protein subunit [15]. The semi-redundant budding yeast Tor1 and Tor2 proteins (mTOR in mammals), Kog1 (regulatory-associated protein of TOR (Raptor) in mammals), and Lst8 (mammalian synthetic lethal with SEC13 protein 8 (mLST8)) form the essential core components of TORC1 [9,16,17,18].

TORC1 is controlled by different growth-related signals, among which amino acids represent primeval cues that are sufficient to activate TORC1 in unicellular organisms such as budding yeast. In multicellular organisms, however, amino acids control TORC1 in parallel to and coordinately with growth factor and hormonal signals. The latter impinge on TORC1 mainly via the Tuberous Sclerosis Complex (TSC), a GTPase activating protein (GAP) complex for the small GTPase Rheb that functions as an essential TORC1 activator in mammals [19,20,21,22,23]. How amino acids regulate TORC1, in contrast, has long remained a mystery. The recent discovery of the Rag GTPases as central elements of an amino acid signaling cascade that impinges on TORC1 in yeast, flies, and mammalian cells, however, represented a breakthrough in this field. This has led to the elucidation of an intriguingly refined system that enables cells to sample the presence of diverse amino acids and fine-tune TORC1 accordingly. Here, we review the respective knowledge of the Rag GTPase signaling network in budding yeast with a special focus on structural aspects of the Rag GTPases and their regulators and a discussion of the evolutionary conservation and divergence of the currently known mechanisms that control Rag GTPases.

## 2. The Rag GTPase Module

**Figure 1:**
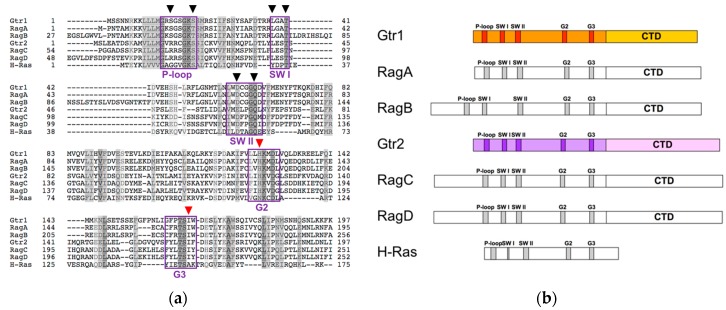
(**a**) Alignment of the G domain sequences of budding yeast (Gtr1 and Gtr2) and human (RagA, RagB, RagC, and RagD) Rag GTPases together with the G domain of human Ras (H-Ras). Conserved residues are highlighted in grey. Boxes denote typical GTPase sequence motifs (see text). Arrow heads mark important residues discussed in the text. Red arrow heads highlight typical Rag GTPase residues; (**b**) Schematic representation of the sequences of yeast and human Rag GTPases together with H-Ras. The sequences were aligned with respect to their G domains.

Gtr1 and Gtr2 define the Rag family of Ras-related GTP-binding proteins in *S. cerevisiae* and RagA, RagB, RagC, and RagD the one in higher eukaryotes [24,25]. Human RagA is orthologous to Gtr1 (48% identity; 75% similarity) and paralogous to RagB (90% identity with 33 additional residues in the N-terminus of RagB). Human RagC is orthologous to Gtr2 (46% identity; 76% similarity) and paralogous to RagD (81% identity with most of the variability lying in the N- and C-terminal regions of both proteins) [25,26,27]. Rag GTPases exhibit all sequence elements that are typically found in GTPases, such as the P-loop (aka PM1) and the switch regions I (SW I; aka PM2) and II (SW II; aka PM3), which are known to mediate phosphate and magnesium binding in GTPases of the Ras family of proteins [25,27,28]. They also contain the guanine base-binding motifs G2 and G3, which, however, diverge from the ones in Ras family proteins due to the presence of a histidine instead of an asparagine in G2 and an isoleucine instead of an alanine residue in G3 (Figure 1a) [24,25,27,29]. These differences in the G2 and G3 motifs originally served to distinguish the Rag GTPases as a separate family of proteins [24,27]. Additional idiosyncratic traits of Rag GTPases include their largely extended C-terminal domains (CTDs) and their lack of lipid modification motifs that typically serve to anchor Ras family proteins to membranes (Figure 1b) [25,30]. Interestingly, their extended C-termini function in the assembly of heterodimeric Rag GTPase complexes that contain Gtr1 and Gtr2, or RagA or RagB combined with RagC or RagD [25,26]. Amino-acid availability promotes a TORC1-activating Rag GTPase module configuration in which Gtr1 or RagA/B is GTP-loaded and Gtr2 or RagC/D is GDP-loaded. Conversely, amino-acid starvation favors the opposite GTP/GDP-loading status within the respective heterodimers, which then inhibit TORC1 [31,32,33,34,35,36]. This setting is unique among GTPases, which are typically active when loaded with GTP and inactive when loaded with GDP [37]. Rag GTPases associate with the Ego1/Meh1–Ego2–Ego3/Slm4 ternary complex (EGO-TC) in yeast or the Ragulator complex in mammals (see below), both of which are predominantly and constitutively anchored within vacuolar or lysosomal membranes, respectively [31,38,39]. In mammalian cells, the active Rag GTPase module recruits TORC1 (via Raptor) to the lysosome where it interacts, in a microspherule protein 1 (MCRS1)-sustained manner [40], with GTP-bound Rheb that stimulates TORC1 through a mechanism that is incompletely understood [34,35,38]. Following amino acid deprivation, in contrast, Rag GTPase heterodimers favor the release of TORC1 from lysosomal membranes due to both their diminished affinity towards TORC1 and their recruitment of TSC that favors the conversion of Rheb^GTP^ to Rheb^GDP^ [32,41]. Like in mammalian cells, Gtr1^GTP^–Gtr2^GDP^ heterodimers, but not the Gtr1^GDP^–Gtr2^GTP^ ones, interact with and activate TORC1 in yeast [31]. However, yeast TORC1 co-localizes with Rag GTPases at the vacuolar membrane and within perivacuolar foci even in the absence of amino acids (or nitrogen) [31,36,42]. Thus, active budding yeast Rag GTPases are not strictly required to tether TORC1 to membranes, although it appears that they may influence the relative distribution of TORC1 between vacuolar membranes and perivacuolar foci under some conditions [43]. Because there is no evidence that suggests a role for the Rheb-orthologous yeast Rhb1 in controlling TORC1, it remains currently unknown how Rag GTPases activate (or inactivate) TORC1 in budding yeast.

### 2.1. Structure and Function of the C-Terminal Domains of Gtr1 and Gtr2

**Figure 2:**
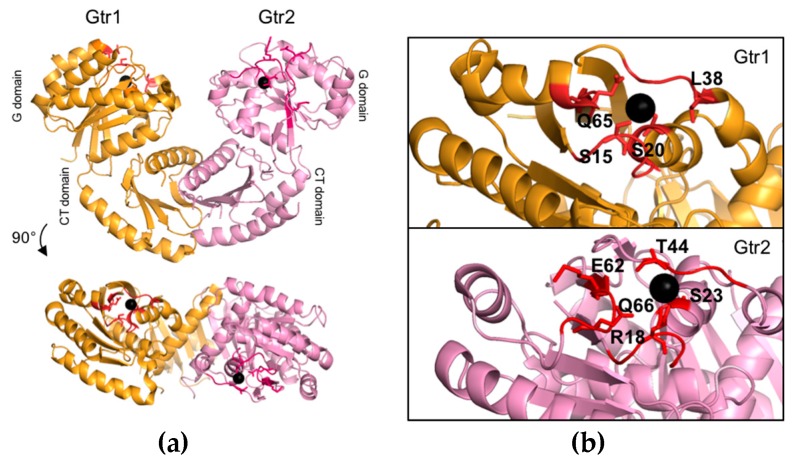
(**a**) Cartoon representation of the structure of the heterodimeric Gtr1–Gtr2 complex ([26], PDB entry 3R7W); (**b**) Details of the G domains of Gtr1 and Gtr2. The red regions correspond to the P-loop and the SW I/SW II domains in each protein. Important residues discussed in the text or listed in Table 1 are labeled. The Mg^2+^ atom is shown as a black sphere.

The structures of mammalian Rag GTPases have not been resolved to date. However, given the high sequence similarities between Gtr1, RagA, and RagB, as well as the ones between Gtr2, RagC, and RagD [25,26], and the fact that functionally critical residues have been evolutionarily conserved between yeast and mammalian Rag GTPases (Table 1), Gtr1 and Gtr2 represent bona fide models to study structural and functional aspects of this family of proteins. In this context, the structures of two different forms of the Gtr1–Gtr2 complex (i.e., Gtr1^GTP^–Gtr2^GTP^ and Gtr1^GTP^–Gtr2^GDP^) have recently been resolved (Figure 2a). Accordingly, both Gtr1 and Gtr2 are part of a complex that adopts a pseudo-twofold symmetry [26,44]. For each subunit two physical domains can be defined: the N-terminal GTPase domain (or G domain) that is responsible for guanine nucleotide-binding and the CTD. The two GTPases dimerize through their CTDs while their G domains are not involved in heterodimer formation [26]. As a result, the dimer interface is distant from the nucleotide-binding pocket, which defines a new architecture that has not yet been described in any other structure of known GTPases (Figure 2a). Of note, other GTPases that have also been described to dimerize typically do so via their G domains [45,46,47,48]. The CTDs of Gtr1 and Gtr2 are structurally very similar and characterized by a central five-stranded anti-parallel β-sheet with a 2-1-4-5-3 topology, surrounded by one long helix on the side of the G domain, and two helices on the opposite side [26]. The heterodimer formation is mediated by hydrogen bonds and hydrophobic interactions mainly involving the α8 helix of Gtr1 that interacts with the α8 helix and two β strands of Gtr2. Conversely, the α8 helix of Gtr2 interacts with the α8 helix and four β strands of Gtr1 [26]. Interestingly, the conserved leucine 207 (Leu^207^) residue located within the β7 strand of both CTDs is of central importance for Rag GTPase dimerization. In line with this notion, substitution of this residue in Gtr1 or Gtr2 with a proline is sufficient to abolish the capacity of Rag GTPases to form heterodimers and to properly assemble with the EGO-TC subunits Ego1 and Ego3 [29]. When expressed within cells, both the Gtr1^L207P^ and Gtr2^L207P^ alleles also cause rapamycin sensitivity, which indicates that Rag GTPase heterodimerization and assembly with the EGO-TC is necessary for proper TORC1 regulation in vivo [29]. In addition to mediating dimerization, the Gtr2-CTD further contributes to an inter-domain interaction and stabilization of the G domain specifically in Gtr2^GDP^ by placing the isoleucine 214 (Ile^214^) into a hydrophobic pocket that is created by the α6 and α1 helices of the respective G domain [44].

### 2.2. Structure and Function of the Gtr1 G Domain

The G domains of Gtr1 and Gtr2 are structurally and functionally much more divergent than their CTDs [26,44]. The G domain in Gtr1^GTP^ is, similarly to other Ras-related GTPases, composed of six α helices, six β strands, two switch regions that interact with the γ-phosphate of GTP via hydrogen bonds, and a Mg^2+^ ion in the nucleotide-binding site [44] (Figure 2b). Biochemical experiments have demonstrated that Gtr1, like Rag A, exhibits an extremely low intrinsic GTP hydrolysis rate when compared to that of other small GTPases like G_i_α_1_ or Ras [27,49,50], which may be explained by at least two structural differences. Firstly, Gtr1, like members of the Arf GTPase family, is missing an important tyrosine (Tyr) in the switch I region that is otherwise conserved in Ras, Rho, and Ran GTPases [44]. The respective Tyr^32^ in Ras plays an important role in stabilizing, via its hydroxyl group, the transition state of the GTP to GDP hydrolysis reaction, which is why mutation of Tyr^32^ to phenylalanine (Phe) decreases the GTP hydrolysis rate of Ras more than 2-fold [51]. The corresponding leucine (Leu) residue in Gtr1 (i.e., Leu^38^), however, cannot favor GTP hydrolysis as it makes no contact with GTP [44]. Secondly, the serine 15 (Ser^15^) in the P-loop of Gtr1 forms a hydrogen bond with the γ-phosphate of GTP, which is not the case for most members of the Ras subfamily that have a glycine (Gly) at the respective position [44]. Ser^15^ is likely to cause a stereochemical constraint that disfavors GTP hydrolysis by Gtr1. This is supported by the findings that mutation of the equivalent serine (Ser^31^) in Rab3A increases its GTPase activity [52], while mutation of the respective Gly residue in Ras (Gly^12^) to Ser creates an oncogenic allele with reduced GTPase activity [53]. Thus, in vivo, Gtr1 is likely predominantly bound to GTP unless its GTP hydrolytic activity is stimulated by a GAP (see below).

Based on a comparison with mutant alleles of Ras, glutamine 65 (Gln^65^) in the switch II region of Gtr1 has also been proposed to be key for GTP hydrolysis [24]. The corresponding residue in Ras (Gln^61^) plays a role in stabilizing, together with Tyr^32^, the transition state of the GTP hydrolysis reaction. Mutation of this residue compromises the GTP hydrolytic activity of Ras, which renders the respective “GTP-locked” Ras alleles oncogenic [51,53]. Similarly, expression of a Gtr1^Gln65Leu^ allele also causes TORC1 hyperactivation and prevents TORC1 inactivation that is mediated by overexpression of the Gtr1 GAP Iml1 (see below) in vivo [31,54]. Together with the observations that the GTP hydrolytic activity of Gtr1 (when combined with Gtr2^Gln66Leu^), but not that of Gtr1^Gln65Leu^ (when combined with Gtr2), can be stimulated by Iml1 [55], these data indicate that the Gtr1^Gln65Leu^ allele is indeed predominantly locked in its GTP-bound form as expected.

Yet another comparison with Ras suggests that mutation of serine 20 (Ser^20^) in the P-loop of Gtr1 may yield a Gtr1 protein that is constitutively inactive [24]. The corresponding serine 17 (Ser^17^) in Ras assists in the coordination of the Mg^2+^ ion that is important for nucleotide binding, and mutation of Ser^17^ in Ras can strongly decrease its affinity for nucleotides (particularly strongly for GTP). This causes the mutated Ras (e.g., Ras^Ser17Ala^ or Ras^Ser17Asp^) to be either nucleotide-free or preferentially GDP-loaded within cells and to function as dominant negative variants of Ras [56,57,58]. In line with the expectation that mutation of Ser^20^ in Gtr1 may similarly affect its biophysical properties, expression of a corresponding Gtr1^Ser20Leu^ allele strongly inhibits growth and acts as a semi-dominant inhibitor of TORC1 in cells [31].

### 2.3. Structure and Function of the Gtr2 G Domain

Structural data with both crystals, namely Gtr1^GTP^-Gtr2^GTP^ and Gtr1^GTP^-Gtr2^GDP^, revealed that Gtr2^GTP^ adopts a similar fold to Gtr1^GTP^ (see above), but significantly changes its conformation when bound to GDP [26,44]. Accordingly, the most dramatic conformational changes take place within the switch I and II regions, which rearrange in a way that Gtr2^GDP^ is composed of only five α helices and five β strands and cannot appropriately coordinate the Mg^2+^ ion in its nucleotide-binding pocket [44]. Because Mg^2+^ mediates many interactions within the pockets of GTPases, its absence is predicted to dramatically increase the GDP dissociation rate [59,60]. This structural feature of Gtr2 therefore explains why it has a relatively low affinity for GDP [44,61], which is also the case for RagC that binds predominantly GTP and releases GDP swiftly [25]. Notably, because GTP is generally much more abundant than GDP within cells [62,63], it is, therefore, possible that Gtr2 (and RagC/D) may not necessarily depend on GTP exchange factors (GEFs) for being reloaded with GTP.

Another distinct feature of the G domain of Gtr2 is that it has, like RagC/D, an arginine in position 18 (Arg^18^) within its P-loop. This residue corresponds to Ser^15^ in Gtr1, which likely hampers GTP hydrolysis by Gtr1 (see above). The structure of Gtr2^GMPPNP^ shows that Arg^18^ in Gtr2 is close to the γ phosphate of GMPPNP and, in contrast to Ser^15^ in Gtr1, likely stabilizes the transition state of the hydrolysis reaction [44]. An analogous Arg residue is also present in the switch I region of G_tα_ where it stabilizes the transition state of the hydrolysis reaction [64]. This specific Arg at position 18 could therefore confer some low intrinsic GTPase activity to Gtr2, as it was observed in Rag C [25,44].

Like Gln^65^ and Ser^20^ in Gtr1, the corresponding Gln^66^ and Ser^23^ residues in Gtr2 are also predicted to be important for GTPase activity and GTP binding, respectively. Mutations of the corresponding residues in Gtr2, however, are expected to have the opposite effect on TORC1 in vivo when compared to the ones in Gtr1. This is indeed the case for Gtr2^Gln66Leu^, the expression of which inhibits TORC1 and consequently also growth [31]. Expression of the Gtr2^Ser23Leu^ allele, on the other hand, does not significantly activate TORC1. This may relate to the finding that Gtr2^Ser23Leu^ stimulates the GTP hydrolysis by Gtr1 in vitro, which could translate into lower levels of active Gtr1^GTP^ in vivo [54]. In support of this model, expression of Gtr2^Ser23Leu^ can significantly activate TORC1 in the presence of an active Gtr1^Gln65Leu^ allele or in the absence of the Gtr1 GAP Iml1 [31,54].

### 2.4. The Gtr1–Gtr2 Module and its Interactions with TORC1

**Figure 3:**
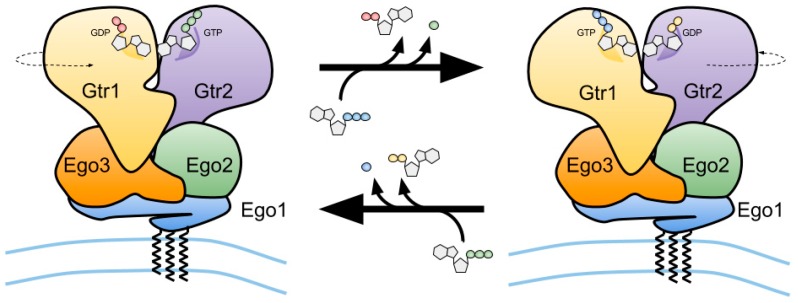
Schematic representation of the vacuolar membrane-bound budding yeast Ego1–Ego2–Ego3 ternary complex (EGO-TC), and of the conformational change of the EGO-TC-associated Gtr1–Gtr2 heterodimer following the exchange of GDP for GTP on Gtr1 and GTP hydrolysis by Gtr2 (upper arrow) and vice versa (lower arrow).

During the hydrolysis of GTP to GDP on Gtr2, the Gtr2 G domain rotates by ≈28° and the one of Gtr1 by ≈6°, each relative to their C domains, thereby forming a new interaction surface between the G domains of Gtr1 and Gtr2 [44] (Figure 3). In this configuration, the newly formed contact between the domains does not change the conformation of the G domain in Gtr1^GTP^. Notably, however, the structural similarities between Gtr1 and Gtr2 predict that Gtr1 can also undergo a similar conformational change to Gtr2 upon GTP-to-GDP conversion. It will therefore be interesting to address whether the nucleotide-binding state of one G domain may indirectly influence the one of the other (e.g., by imposing steric constraints that dictate the association/dissociation or hydrolysis of the nucleotide within the other protein). The dynamic structural changes that come along with binding and hydrolysis of GTP within the Gtr1–Gtr2 module likely modulate their interactions with effector proteins such as TORC1. As outlined above, it is the active form of this heterodimer in both yeast (i.e., Gtr1^GTP^–Gtr2^GDP^) and mammals (i.e., RagA^GTP^/B^GTP^–RagC^GDP^/D^GDP^) that preferentially interacts with the TORC1 subunit Kog1 (and Tco89) or Raptor, respectively [26,29,31,35,36,43,65]. Because the regions that are predicted to undergo the most dramatic structural changes upon GTP hydrolysis within the G domain of Gtr1 also correspond to the ones that have been mapped in RagA/B to interact with Raptor [26] (Table 1), it is likely that GTP hydrolysis and the ensuing G domain rotation in Gtr1/RagA/B conceals a binding surface for TORC1. In this context, it is also worth noting that, deduced from the Gtr1^GTP^–Gtr2^GDP^ crystal structure, the TORC1-interacting surface of RagA^GTP^/RagB^GTP^ would form a continuous surface with RagC^GDP^/RagD^GDP^, which could elegantly explain why both RagB and RagC contribute to the binding of Raptor, with the latter having a key role depending on its nucleotide binding status [35,65].

## 3. Regulators of Rag GTPases

**Figure 4:**
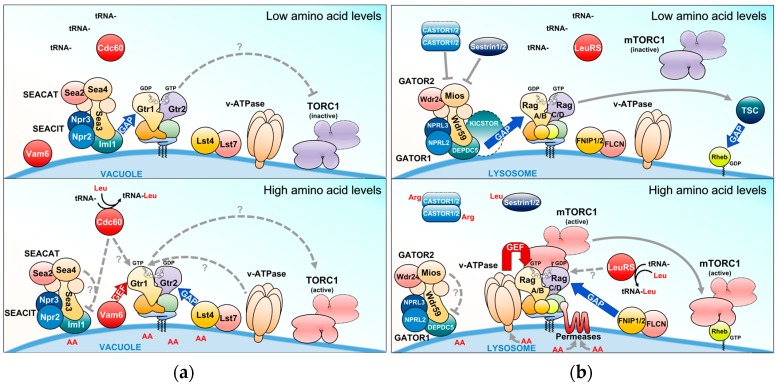
The Rag GTPase signaling network in yeast and mammals. (**a,b**) Upstream regulators that antagonize (when amino acids are limiting; upper panels) or stimulate (when amino acids are abundant; lower panels) the Rag GTPase- target of rapamycin complex 1 (TORC1) signaling branch in yeast (**a**) and mammalian (**b**) cells. Red and blue arrows indicate GTP exchange factors (GEF) and GTPase activating protein (GAP) activities, respectively. Arrows and bars denote activating and inhibiting activities, respectively. Dashed arrows and question marks indicate mechanisms that are currently only partially understood. For further details, see text.

Rag GTPases mediate amino acid signals towards TORC1 and their GTP/GDP-loading status is a critical determinant in this process. Research in this field has therefore more recently focused on the identification of the regulators of the nucleotide-binding state of Rag GTPases (such as GAPs and GEFs) and on the elucidation of the sensory mechanisms that mediate amino acid signals to these regulators (Figure 4). The following sections provide a brief overview of the recent advances in these endeavors.

### 3.1. The EGO-TC and Mammalian Ragulator

Unlike other small GTPases such as Ras, Gtr1 and Gtr2 are not modified by lipid moieties that could mediate their anchoring to membranes. The Gtr1–Gtr2 module, however, is recruited to the vacuole via its binding to a ternary complex (coined EGO-TC), which consists of Ego1, Ego2, and Ego3 [31,33,39,66]. All five proteins (Gtr1, Gtr2, Ego1, Ego2, and Ego3) form the EGO complex (EGOC), the name of which originated from a screen for mutants that exhibited a defect in exit from rapamycin-induced growth arrest [66]. Similar to EGO-TC, mammalian Rag GTPases are tethered to lysosomes by a protein complex coined Ragulator [38]. This complex is composed of five different proteins: lysosomal adaptor and mitogen-activated protein kinase and mTOR Ragulator 1 (LAMTOR1)/p18, LAMTOR2/p14, LAMTOR3/MP1, LAMTOR4/C7orf59, and LAMTOR5/HBXIP [67]. Although the sequence similarity between any of the different subunits within EGO-TC and Ragulator is low, structural comparisons indicate that EGO-TC is likely the equivalent of Ragulator [39]. For instance, Ego2/3 and LAMTOR2-5 all contain a roadblock domain, which is defined by an α-β-α sandwich with the central β-sheet flanked by one α-helix on the bottom and one or two α-helices on the upper side. Due to differences in the amount of the flanking α-helices, EGO-TC and Ragulator subunits can be subdivided into two different groups containing either Ego2, LAMTOR4, and LAMTOR5 (group I) or Ego3, LAMTOR2, and LAMTOR3 (group II; with an additional α-helix on the upper side compared with group I proteins) [39,68]. Due to its N-terminal myristoylation and palmitoylation [69,70], Ego1 functions as a tether for the entire EGOC [31] (Figure 3). Similarly, LAMTOR1 tethers the Rag GTPase-Ragulator complex to the lysosomal surface via its N-terminal palmitoyl- and myristoyl-moieties and functions as a scaffold for the other subunits [67,71]. While the EGO-TC plays primarily a role as a platform that recruits the Gtr1–Gtr2 heterodimer to the vacuolar membrane [39], the role of Ragulator appears not to be restricted solely to scaffolding the Rag GTPase to the lysosomal membrane. Instead, Ragulator also functions as a GEF for RagA/B downstream of the vacuolar H^+^-ATPase (v-ATPase) [67] (see also below). While there is currently no data available that suggest a role for EGO-TC as a GEF for Gtr1, this task is exerted by Vam6 (alone or combined with additional associated proteins) in yeast [31,68,72] (Figure 4a). Vam6 (aka Vps39) is a component of the homotypic fusion and vacuole protein sorting (HOPS/Class C-Vps) complex that promotes vacuolar fusion events as an effector of the Rab7 GTPase Ypt7 [73,74,75]. As such, the HOPS complex is also required for proper TORC1 signaling [76,77], but Vam6 clearly plays additional HOPS complex-independent roles. For instance, Vam6 is specifically involved in the establishment of contact sites between vacuoles and mitochondria to facilitate the lipid transfer between these organelles [78,79]. Vam6 therefore occupies a central stage in coordinating cellular events such as membrane fusion, lipid exchange, and metabolism with TORC1 activity. Notably, higher eukaryotes express two Vam6-like proteins, namely hVps39-1 (or hVam6) and hVps39-2 [80,81,82], of which Vps39-2 is orthologous to the class C core vacuole/endosome tethering (CORVET) complex subunit Vps3 [83]. Knockdown of hVps39-1 blocks early-to-late endosome conversion and reduces mTORC1 activity [84]. Nevertheless, hVps39-1 does not function as a GEF for RagA/B, since it does not stimulate GDP or GTP dissociation from RagB in vitro and does not bind RagA in vivo [67].

### 3.2. SEACIT and SEACAT and the Orthologous Mammalian GATOR Complexes

Genetic experiments in yeast identified Npr2 and Npr3 as negative regulators of TORC1 [85]. Both proteins are part of the octameric Seh1-associated protein complex (SEAC) [86], and form, together with Iml1, the trimeric SEACIT subcomplex (for SEAC inhibiting TORC1) that antagonizes TORC1 by acting as a GAP module on Gtr1 [54,87]. Interestingly, the remaining five proteins (Seh1, Sec13, Rtc1/Sea2, Mtc5/Sea3, and Sea4) form another SEAC subcomplex termed SEACAT (for SEAC activating TORC1) that stimulates TORC1 likely by inhibiting SEACIT via a mechanism that is currently not understood [87]. SEACIT localizes at the vacuolar membrane where Iml1 transiently stimulates the GTPase activity of Gtr1 in an Npr2-/Npr3-dependent manner when cells are starved for amino acids [87] (Figure 4a). How amino acids impinge on SEACIT/SEACAT is currently not known. However, methionine favors SEACIT disassembly through biosynthesis of S-adenosylmethionine, which serves as methyl donor for Ppm1-mediated methylation and activation of the catalytic subunit of the type 2A protein phosphatase (PP2A). The latter dephosphorylates Npr2 to prevent it from associating with Npr3 and Iml1 [88].

SEACIT and SEACAT have functionally and structurally related orthologs in mammals named GATOR1 (GAP activity towards Rags; containing DEPDC5, NPRL2, and NPRL3) and GATOR2 (containing Seh1L, Sec13, Wdr24, Wdr59, and Mios), respectively [89,90]. Like their yeast counterparts, GATOR1 functions as RagA/B GAP to inactivate TORC1 when amino acids are scarce, while GATOR2 likely inhibits GATOR1 when amino acids are present abundantly (Figure 4b, see also below; of note, the GATOR2 subunit Wdr24 also controls lysosome acidification and autophagic flux independently of TORC1 in fly and mammalian cells [91]). The recruitment of GATOR1 to the lysosomal surface and its interaction with Rag GTPases requires the KPTN, ITFG2, C12orf66 and SZT2-containing regulator of TORC1 (KICSTOR) scaffolding complex, and loss of KICSTOR components (that are not readily identifiable in yeast) leads to hyperactive TORC1 [92,93]. The GATOR1-RagA association is additionally regulated by ubiquitination. Amino acid starvation, for instance, stimulates the respective association by K^63^-linked ubiquitination of RagA on Lys^142^, Lys^220^, Lys^230^ and Lys^244^ via the RING family E3 ligase RNF152. This serves to inactivate RagA and consequently also TORC1 [94]. Similarly, ubiquitination of Lys^15^ in RagA by the Skp1/Cullin/F-box E3 ligase complex also promotes the GATOR1-RagA interaction, although in this case this happens during amino acid refeeding as part of a feedback loop that prevents TORC1 hyperactivation [95]. Whether analogous ubiquitination mechanisms regulate the interaction of SEACIT with Gtr1 in yeast remains to be studied, but at least Lys^230^ and Lys^244^ appear to be conserved in Gtr1 (Table 1).

### 3.3. The Lst4–Lst7 and Orthologous FNIP1/2–FLCN Complexes

FNIP1 and FNIP2 each combine with Folliculin (FLCN) to form a complex that is required for the amino acid-mediated recruitment of TORC1 to the lysosome by the Rag GTPases [65,96]. Interestingly, although the FNIP1/2–FLCN module directly and preferentially binds the TORC1-inactivating versions of RagA/B in starved cells [65,96,97], it stimulates the GTPase activity of RagC/D and hence promotes the binding of Raptor with the Rag GTPase heterodimer at the lysosomal membrane [65] (Figure 4b). It is, therefore, conceivable that the FNIP1/2–FLCN complex remains tethered to the lysosomal membrane in the absence of amino acids only to be properly placed and ready to stimulate GTP hydrolysis by RagC/D. FNIP1/2–FLCN thus favors the recruitment of TORC1 to the lysosomal surface where it can be activated by Rheb, when amino acids become available. Yeast express a FNIP1/2–FLCN-orthologous complex, namely Lst4–Lst7, that functions as GAP for Gtr2 specifically when starved cells are refed with amino acids [55] (Figure 4a). Similar to FNIP1/2–FLCN, the Lst4–Lst7 complex also accumulates at the vacuolar membrane in amino acid-starved cells and is dispersed form this location when cells are refed with amino acids. At variance with the situation in higher eukaryotes, however, where the FNIP1/2–FLCN heterodimer binds Rag GTPases in starved cells, the Lst4–Lst7 complex binds Rag GTPases weakly in the absence of amino acids, but more strongly in amino acid-fed or re-fed cells. In addition, rather than binding Gtr1 or GDP-free Gtr1^Ser20Leu^, the Lst4–Lst7 complex favors binding GTP-locked Gtr2, which is a trait that is common for GTPases and their cognate GAPs [55]. Thus, the Lst4–Lst7 complex associates with the vacuolar membrane proximal to, but not via the Rag GTPases in amino acid starved cells. There, it is able to stimulate the GTPase activity of Gtr2 upon amino acid refeeding, which results in TORC1 activation and release of Lst4–Lst7 from the vacuolar membrane. In this context, it will be interesting to explore whether the latter effect is part of a feedback inhibition loop that may serve to protect TORC1 from hyperactivation upon amino acid refeeding. Notably, methionine, cysteine, glutamine, as well as asparagine and aspartate that can be deaminated and converted to glutamate/glutamine [98], are all specifically efficient in displacing the Lst4–Lst7 complex from the vacuolar membrane. How these amino acids are sensed and how the respective sensory mechanisms impinge on Lst4–Lst7 are therefore key questions to be addressed in the near future.

## 4. Amino Acids and Their Sensors Upstream of the Rag GTPases

Despite the fact that wild-type budding yeasts are prototrophic, i.e., can synthesize all amino acids on their own, they can sense the presence of any extracellular amino acid and mediate the respective signal to activate TORC1 within minutes, although with different amplitudes [99]. The underlying common or specific sensory mechanism(s) are currently, in most cases, still elusive. Similarly, it is not known whether all of the respective signals regulate TORC1 via the Rag GTPases, but many (e.g., methionine, cysteine, glutamine, asparagine, and aspartate) clearly do so [55,88,99,100]. In this context, it is worth noting that yeast cells can also use virtually any amino acid as nitrogen source, but the different amino acids vary greatly with respect to their capacity to sustain vigorous growth. Accordingly, yeasts preferentially grow on high quality nitrogen sources such as arginine, asparagine, and glutamine, while they grow for instance very poorly on the branched-chain amino acids leucine, isoleucine, and valine [98]. Importantly, the quality of the amino acid as a nitrogen source, or its metabolic input value, is also coupled to TORC1, albeit via Rag GTPase-independent mechanisms that ultimately define the relative growth rate of cells. Thus, any given amino acid potentially contributes in qualitatively, quantitatively, temporally, and mechanistically distinct ways to TORC1 regulation. Adding to this complexity, some amino acids have become essential (e.g., branched-chain amino acids) or conditionally essential (e.g., arginine and glutamine) in higher eukaryotes including humans, which may have favored the evolution of specific amino acid-sensory system that needed to be newly wired to TORC1. In the following, we provide a brief overview on our current knowledge of specific amino acids that have recently been discovered to be coupled to the control of the Rag GTPase-TORC1 branch.

### 4.1. Leucine

The branched-chain amino acid leucine is the most frequently encoded amino acid in eukaryotic genomes [101]. It is, therefore, not surprising that leucine also activates TORC1 [102]. Two studies have provided insight into how the levels of leucine, or of branched-chain amino acids, are sensed and transmitted to Rag GTPases. Accordingly, a classical co-IP approach pinpointed the leucyl-tRNA synthetase (LeuRS) Cdc60 as a leucine-dependent, Gtr1-interacting protein [103]. LeuRS is both necessary and sufficient to mediate leucine signaling to Gtr1 and this positive input is disrupted by the engagement of LeuRS in editing mischarged tRNA^Leu^, which is likely a consequence of leucine limitation. In the presence of leucine, however, LeuRS interacts with and promotes the GTP-loading status of Gtr1, likely by protecting Gtr1 from a GAP and/or by assisting a positive regulator (Figure 4a). LeuRS therefore senses the balanced levels of branched-chain amino acids via the fidelity of tRNA^Leu^ aminoacylation and signals this information via an incompletely understood mechanism to the Rag GTPases. This also fits well with the finding that branched-chain aminotransferases, which interconvert leucine α-ketoisocaproate and leucine, act upstream of the LeuRS-EGOC-TORC1 signaling module, although they also control TORC1 in parallel by controlling the flux through the tricarboxylic acid (TCA) cycle via their interaction with the key TCA-cycle enzyme Aco1 [100]. Strikingly, mammalian LeuRS has also been found to control TORC1, both by Rag GTPase-dependent and -independent mechanisms [104,105]. The former requires LeuRS itself to function as a RagD GAP when leucine is present [104] (Figure 4b), even though this was not recapitulated in an independent study [65].

Leucine has recently also been discovered to feed into the Rag GTPase network via a group of paralogous proteins coined Sestrins [106,107,108,109,110]. Intriguingly, Sestrin2 directly binds leucine and a crystal structure could be obtained that reveals both a leucine-binding pocket and a highly conserved GATOR2 binding site [111]. Moreover, Sestrin2 (and to a lesser extent Sestrin1 and 3) interacts with GATOR2 predominantly during amino acid deprivation to inhibit TORC1 indirectly via GATOR1 [106,107,108] (Figure 4b). It is, therefore, conceivable that Sestrin2 antagonizes GATOR2 when it is not bound to leucine and that leucine-binding triggers a conformational change in Sestrin2 that masks its GATOR2 binding site [111]. However, whether Sestrin2 indeed undergoes such a conformational change is currently a matter of debate as it appears that the leucine-free apo-structure of Sestrin2 is still elusive [112,113,114]. Notably, one study also suggested that Sestrins regulate Rag GTPases more directly by acting as GDP dissociation inhibitors (GDIs) of RagA/B, which would inactivate the Rag GTPase heterodimer and consequently TORC1 under amino acid-starvation conditions [109]. Thus, Sestrin2 may do both, act directly and indirectly (via GATOR2) on Rag GTPases. However, because the GDI motif is buried within the structure of the leucine-bound form of Sestrin2, this would require Sestrin2 to adopt a different conformation in the absence of leucine such that the respective motif would be exposed at the surface of the protein [111]. Finally, Sestrins are also stress-inducible proteins that inhibit TORC1 through the AMP-activated protein kinase (AMPK) and the TSC complex even under amino acid-replete conditions [114,115]. It appears therefore that Sestrins, which are not readily identifiable in yeast, have evolved to control TORC1 via multiple different mechanisms.

### 4.2. Glutamine

Glutamine is of central importance for supplying carbon and nitrogen atoms for biosynthetic reactions (e.g., biosynthesis of amino acids, nucleotides, and the primary cellular antioxidant glutathione) and for replenishing the TCA cycle, hence providing the bioenergy to drive cellular growth (for a review see [116]). Current knowledge indicates that glutamine regulates TORC1 via both Rag GTPase-dependent and -independent ways, although the mechanistic details remain poorly understood in each case. In mammals for instance, glutaminolysis, i.e., the sequential deamination of glutamine by glutaminase (GLS) and glutamate dehydrogenase (GDH), stimulates α-ketoglutarate production and consequently activation of the Rag GTPase-TORC1 branch through an incompletely resolved mechanism that implicates the prolyl-hydroxylase PHD [117]. Of note, leucine also contributes in this framework to glutamine-mediated TORC1 activation by acting as a co-factor for GDH [117,118,119]. Conversely, glutamine contributes to leucine-mediated TORC1 activation by acting as an anti-solute to import leucine into the cytoplasm via the SLC7A5–SLC3A2 heterodimeric antiporter [120], which also plays a role upstream of TORC1 by modulating the distribution of glutamine and leucine between the lysosome and the cytoplasm [121]. Glutamine activates TORC1 also independently of the Rag GTPases in both yeast and mammalian cells [99,122]. In mammalian cells, this requires the ADP-ribosylation factor 1 (Arf1) and the v-ATPase [122]. Whether yeast may also employ Arf1 to control TORC1 is not known, but recent evidence indicates that the vacuolar membrane-associated phosphatidylinositol 3-phosphate binding protein Pib2 acts together with a Vps34–Vps15 phosphatidylinositol 3-kinase complex to mediate glutamine signals to TORC1 in parallel to EGOC [123,124,125].

### 4.3. Arginine

How yeast TORC1 integrates arginine signals is presently unknown. In higher eukaryotes, however, arginine activates TORC1 through at least two different mechanisms. The first one involves the lysosomal membrane-localized amino acid transporter SLC38A9, which communicates (likely lysosomal) arginine levels to the Rag GTPases to control TORC1 [126,127,128]. SLC38A9 associates with both Ragulator and the Rag GTPases in an amino-acid dependent manner and loss of SLC38A9 compromises arginine-induced TORC1 activation. The second mechanism relies on the cytosolic arginine sensor CASTOR1 that forms homo- or heterodimers (with CASTOR2), which, like Sestrin2, bind and inhibit GATOR2 in the absence of arginine [129]. Arginine binding disengages CASTOR dimers from binding GATOR2, which may thereby become competent to inhibit GATOR1 and consequently stimulate TORC1 (Figure 4b). Structural analyses of homodimeric CASTOR1 revealed that this mechanism relies on arginine binding between two (of four) ACT (for aspartate kinase, chorismate mutase, and TyrA) domains, which likely triggers a conformational change that conceals an adjacent binding surface for GATOR2 (or more specifically for its subunit Mios) [130,131,132]. Interestingly, although yeast cells do not express CASTOR proteins [12], CASTOR1 may have evolved from the regulatory domain of ancestral aspartate kinases including Hom3 in yeast [130]. Together with the finding that loss of Hom3 renders cells sensitive to rapamycin and exhibits negative genetic interactions with loss of SEACAT (i.e., Seh1, Sec13, and Mtc5/Sea3) and EGOC (i.e., Ego1 and Ego3) subunits [133,134], this warrants further analyses that address the possibility that Hom3 may also mediate amino acid signals to TORC1 via the Rag GTPases.

### 4.4. The V-ATPase and Vacuolar/Lysosomal-Membrane Resident Amino Acid Permeases

The amino acid-sensitive branch of TORC1 signaling localizes at the vacuolar/lysosomal periphery where it is ideally placed to integrate both cytosolic and vacuolar/lysosomal amino acid pools. Because amino acids (and other metabolites) shuttle across the vacuolar/lysosomal membrane through amino acid permeases that function either as H^+^-antiporter (in) or H^+^-symporter (out) [98,135], their distribution between the two compartments is largely driven by the proton gradient across the respective membrane. The latter is established through the vacuolar/lysosomal H^+^-ATPase (v-ATPase), a proton-pump that hydrolyses ATP to import protons into the vacuolar/lysosomal lumen [136,137]. The v-ATPase therefore plays a key role in controlling TORC1 both indirectly via its effect on the subcellular distribution of amino acids, but also through its role in pH homeostasis [138,139]. Moreover, in flies and mammals, the v-ATPase also directly interacts with the Ragulator-Rag GTPase complex to promote the GEF activity of Ragulator toward RagA/B in response to intra-lysosomal amino acids [67,140] (Figure 4b). How and which amino acids signal through the v-ATPase remains, however, still elusive. In yeast, the v-ATPase also acts upstream of the Rag GTPases, but it controls TORC1 in response to the pH in the cytoplasm, which serves as a proxy for the quality and quantity of the available carbon source [141]. Whether the yeast v-ATPase-Rag GTPase module is implicated in signaling vacuolar amino acid levels has not yet been reported.

In addition to SLC38A9, other lysosome-based amino acid permeases have also been found to control TORC1. For instance, the proton and amino acid symporter PAT1/SLC36A1, which shuttles small unbranched amino acids across the lysosomal membrane, is required for mTORC1 activation by amino acids and interacts with RagC/D [142,143]. Similarly, SLC15A4 is a lysosomal proton-coupled histidine transporter, which mediates TORC1 activation during the inflammatory response [144]. Although it remains unclear whether SLC15A4 regulates TORC1 via the Rag GTPases, these studies indicate that the Rag GTPase-TORC1 branch may be poised to integrate information on specific amino acids in part by interrogating a diverse array of vacuolar/lysosomal amino acid permeases in parallel. Because yeast cells express vacuolar membrane-resident amino acid permeases that are structurally and functionally similar to SLC38A9, PAT1/SLC36A1, and SCL15A4 [98], it will be interesting to evaluate whether the emerging concept of amino acid permeases as transceptors that signal amino acid levels to Rag GTPases/TORC1 is of ancestral origin.

## 5. Additional Signals and Modulators that Impinge on Rag GTPases

Besides their role in mediating amino acid signals, the Rag GTPases have also been found to transmit information on other nutrient cues. The mammalian v-ATPase-Ragulator complex, for instance, recruits AXIN, which inhibits the GEF activity of Ragulator and activates AMPK through LKB1 when cells are starved for glucose [145]. Interestingly, expression of constitutively active RagA^GTP^ prevents TORC1 inactivation under similar conditions [146], which indicates that the Rag GTPases mediate also glucose sufficiency in higher eukaryotes. In yeast, glucose starvation also results in TORC1 downregulation, but the role of the Rag GTPases in this process appears less influential [147,148]. In addition to glucose, cholesterol has also recently been found to activate the Rag GTPase-TORC1 branch via the arginine sensor/transporter SLC38A9, which binds lysosomal cholesterol through a conserved cholesterol-binding motif within its transmembrane helix 8 and mediates the respective signal in a manner that is independent of its arginine-sensing mechanism [149]. In parallel, SLC38A9 also binds the Nieman-Pick C1 (NPC1) protein that antagonizes TORC1 activation by shuttling cholesterol back into the cytoplasm.

In addition to their regulation by classical means (that is by GAPs and GEFs), Rag GTPases have also been found to be controlled by a number of different modulators specifically in higher eukaryotes. For instance, the MAP4K3 protein kinase positively regulates TORC1 and physically interacts with and likely acts upstream of RagA and Rag C [150,151,152]. In addition, p62 binds to and promotes the active form of the Rag GTPases to form a docking platform for TORC1 at the lysosomal membrane in response to amino acids [153]. A similar positive role in Rag GTPase-TORC1 control is exerted by the Nudix-type motif 2 (NUDT2) protein [154]. The SH3 domain-binding protein 4 (SH3BP4), in contrast, binds TORC1-inactivating Rag GTPases to inhibit their conversion into the TORC1-activating form and consequently antagonizes the recruitment of TORC1 to the lysosome [155]. Finally, c17orf59 modulates TORC1 activity by interacting with Ragulator and disrupting the Rag GTPase-Ragulator interaction [156]. Whether any of these additional regulatory modules represent ancestral modes of Rag GTPase control is currently not known. Lastly, we would like to point out that several recent studies also indicated the existence of pathways by which amino acids regulate TORC1 independently of Rag GTPases. Although the respective mechanisms are not integral elements of the Rag GTPase signaling network, we would like to refer the reader here to the corresponding primary literature [122,157,158,159,160].

## 6. Final Remarks

The basic architecture of the Rag GTPase-TORC1 signaling network is remarkably conserved within the eukaryotic kingdom [161,162,163], and mutations in individual components of this network are associated with various human pathologies such as immunodeficiency, epilepsy, and cancer [12,164]. Understanding the molecular details by which the Rag GTPases integrate amino acid and other signals in various model systems ranging from yeast, over flies to mammalian cells is therefore indispensable for the establishment of therapies against diseases that are causally related to deregulated TORC1. Pertinent issues that remain to be addressed in this rapidly expanding field of Rag GTPase-centered research are manifold. For instance, structural analyses of the Rag GTPases combined with the pentameric Ragulator, which are currently not available, would help understand how Ragulator associates with and exerts, unlike the functionally orthologous EGO-TC in yeast, GEF activity towards Rag GTPases. Similarly, structural analyses regarding the association of the Rag GTPases with TORC1, the structure of which has also been solved recently [165,166,167,168], are likely to yield a more precise comprehension of the molecular details through which Rag GTPases activate and/or inactivate TORC1. Finally, structural studies of the Rag GTPases combined with their cognate GAPs (i.e., SEACIT/GATOR1 with Gtr1/RagA/B and Lst4–Lst7/FNIP1/2–FLCN with Gtr2/RagB/C) would provide valuable information on the basic mechanisms through which these GAPs stimulate GTP hydrolysis by Rag GTPases. In parallel to all of these structural studies, further biochemical studies (which may perhaps be suitably studied using nuclear magnetic resonance spectroscopy) may address the question whether the Rag GTPases themselves can impact on each other to mutually modulate their GTP/GDP-loading status. Moreover, and beyond these structural/functional issues regarding the core of the Rag GTPase module, it still remains to be queried how exactly the currently known amino acid sensors such as the LeuRS, the lysosomal amino acid permeases, the v-ATPase, and the Sestrin/CASTOR proteins transmit their information to the Rag GTPases. In this context, it appears equally important to decipher how the different signals are gauged and integrated by the Rag GTPases both in qualitative and quantitative terms. Last, but not least, it will be interesting to appropriately define the ancestral Rag GTPase network design to be able to appreciate those mechanisms that have specifically been grafted onto the Rag GTPase module through evolution. Although recent research has dramatically broadened our view on how amino acids impinge on TORC1, this brief, non-comprehensive outline of unsolved questions illustrates that this fascinating field of research still holds many secrets that remain to be unveiled.

## Figures and Tables

**Table 1:** Important residues in Rag GTPases.

**Protein**	**Residue**	**Function**	**Orthologous Site in RagA/RagB**
Gtr1	S15	Possibly impairs GTP hydrolysis	S16/S49
Gtr1	S20	Mg^2+^ coordination	T21/T54
Gtr1	L38	Does not favor GTP hydrolysis	L39/L72
Gtr1	Q65	Stabilizes the transition state	Q66/Q127
Gtr1	L207	Dimerization	L205/L266
**Protein**	**Residue**	**Function**	**Orthologous Site in RagC/RagD**
Gtr2	R18	Stabilizes the transition state	R70/R71
Gtr2	S23	Mg^2+^ coordination	S75/S76
Gtr2	T44	Mg^2+^ coordination	T96/T97
Gtr2	E62	Mg^2+^ coordination	D116/D117
Gtr2	Q66	Stabilizes the transition state	Q120/Q121
Gtr2	L207	Dimerization	L261/L262
Gtr2	I214	G domain stabilization	L268/L269
**Protein**	**Residue(s)**	**Function**	**Orthologous Site(s) in Gtr1**
RagA	R24/S25/N30/Y31	Interaction with Raptor	R23/S24/N29/Y30
RagA	R34/D35/R37/R38	Interaction with Raptor	-/D34/R36/R37
RagA	H47/H49/R51	Interaction with Raptor	H46/H48/R50
RagA	N55/V57/N59/W61	Interaction with Raptor	N54/-/N58/W60
RagA	K230	Ubiquitinated, regulates interaction with GATOR1	K245
RagA	K244	Ubiquitinated, regulates interaction with GATOR1	K259
